# A Five Million Army Means Fifty Thousand Medical Officers

**Published:** 1918-10

**Authors:** 


### A Five Million Army Means Fifty Thousand Medical Officers.

With an army of three million men in the field or in training and, as contemplated, an expansion of this force to five million men, the Surgeon General must have in the Medical Reserve Corps at least fifty thousand doctors.

The Medical Corps must keep a pace in growth with the army expansion, and it behooves every doctor in the United States between the age of 21 and 55, who is physically, morally and professionally fitted, to arrange at the earliest possible moment, his personal affairs so as to offer his services to his country in the capacity of a medical officer.

The United States is in the war to do her part in winning the struggle, and this can only be accomplished by a large and well trained body of troops adequately cared for by sufficient number of medical officers. The importance of the doctor's service and its relation to the successful outcome of the war cannot be over-estimated.

As the mobile forces increase in size, so is there an expansion of base hospitals and other institutions for the care of the sick and wounded, and there should be no lack of officers when required to give to our patriotic boys that professional attention which is so essential.

It is well for the medical profession of the United States to realize at once that a Medical Reserve Corps of at least 50,000 doctors will be required to meet the demands of the Surgeon General and upon which corps he can draw for his medical officers.

We believe by this time that the profession of this country must be fully alive to the needs of the service, so let every doctor who is qualified, feel that he is doing not only his patriotic duty in offering his services as a medical officer, but is relieving the tension of the Surgeon General's office by placing at the command of the Chief Officer of the Medical Department an adequate force without the frequent beating of drums to supply the necessary number with each increase of the mobile forces.

If you have not already received an application blank for commission in the Medical Reserve Corps, your nearest Examining Board or the editor of this journal will be glad to supply you.

---

A special treasury investigating committee reports an increase in the number of drug addicts in the United States and suggests drastic measures for stopping the spread of the habit. The best physicians will gladly render what assistance they can in putting an end to this growing evil, for they recognize that the drug habit is even worse than the appetite for liquor, when once it is firmly established.

---

At this writing the Spanish influenza in the South has been of a mild type and the number of deaths therefrom has been relatively small. Fortunately the whole machinery of the United States Government was brought to bear early to stamp it out, and its spread is being rapidly checked.

